# Viral Cirrhosis: an Overview of Haemostatic Alterations and Clinical Consequences

**DOI:** 10.4084/MJHID.2009.033

**Published:** 2009-12-30

**Authors:** Francesca Romana Ponziani, Valerio De Stefano, Antonio Gasbarrini

**Affiliations:** 1 Department of Internal Medicine and Gastroentrology, Catholic University - Rome; 2 Institute of Hematology, Catholic University - Rome

## Abstract

Viral hepatitis is a major health problem worldwide, the principal cause of cirrhosis and hepatocarcinoma. Once cirrhosis occurs, the consequences of liver dysfunction and portal hypertension become evident and, sometimes, life threatening for patients. Among the various complications of liver cirrhosis, the alteration of haemostatic balance is often a hard challenge for the clinician, since it is capable to predispose both to bleeding or thrombosis. In this review, we analyze the principal aspects of procoagulant, anticoagulant and fibrinolytic capacity of cirrhotic patients, which appears to be variably altered in all these aspects, not only in the direction of a tendency to bleeding. Laboratory investigations, at present, may provide only a partial representation of this condition, because of the impossibility to obtain a test capable to furnish a global overview of the haemostatic system and to reproduce *in vivo* conditions. Furthermore, we describe the pathophysiological mechanisms underlying bleeding manifestations and thrombosis development in cirrhotic patients, which should be considered not only as obvious consequences of the advanced liver disease but, rather, as the result of a complex interaction between inherited and acquired factors.

## Introduction:

In the late 1960’s the only known pathogens belonging to hepatitis virus family were hepatitis virus A and B^1^, the former spread through fecal-oral transmission with short incubation period, the latter spread by blood contamination with a longer incubation period. About ten years later, blood samples from patients with a suspected transfusion-associated hepatitis B, were found to be negative for both B or A virus infection. That was the first identification of “non-A, non-B”, hepatitis C virus^2^. Hepatitis B and C are the most diffused infections worldwide and the principal cause of cirrhosis and hepatocellular carcinoma. About 350 and 180 million people are affected by hepatitis B and C, respectively: viral hepatitis is a major health problem, interesting approximatively 3–6% of world population^3^,^4^,^5^,^6^,^7^. In low income countries of SouthEast Asia and Africa hepatitis B and C are endemic, due to socioeconomic conditions; in contrast, in industrialized countries the burden of hepatitis B and C has been related to the use of infected blood products or other iatrogenic procedures, sexual transmission or drug abuse. Nowadays, prophylaxis and control of medical devices and a specific educational policy have strongly downsized the risk of transmission^8^. By the way, about 8–20% of patients with chronic hepatitis B develop liver cirrhosis within 5 years, while hepatitis C virus is responsible for 40% of cases of endstage cirrhosis in industrialized countries^8^. Viral cirrhosis, except for some histological features, is not clinically different from cirrhosis of other origin. Both viral and non-viral cirrhosis cause impairment in synthetic, catabolic and metabolic function of the liver and lead to portal hypertension, possibly complicated by portal vein thrombosis (Amitrano & Guardascione, in this issue). However, the incidence of hepatocellular carcinoma, is higher among patients with viral cirrhosis, and requires often a strict follow-up and a prompt treatment, whether possible (Granito & Bolondi, in this issue).

In this article, we describe one of the most interesting and debated arguments in matter of liver disease and cirrhosis, the alteration of haemostatic system and the predisposition to hypo- and hypercoagulability. We will analyze the dichotomy between tendency to bleeding and tendency to thrombosis, and its pathophysiologic aspects in cirrhotic patients, reporting perplexities and certainties of a long lasting debate.

## The Haemostatic System in Patients With Liver Cirrhosis:

Bleeding is a common and well-know problem in clinical management of cirrhotic patients. Several conditions, often associated, have been recognized to predispose to a major hemorrhagic event. The principal one is portal hypertension, which predispose to varices development, portal hypertensive gastrophaty, splenomegaly and secondary thrombocytopenia; nevertheless, anticoagulant treatment or excessive liquid infusion may favor bleeding, directly or through dilution of clotting factors. By the way, it is common opinion that in patients with a severe coagulative defect, due to impaired liver function, bleeding occurs only in the presence of anatomic lesions. Recently, this widespread belief has been revised, underlying the profound modifications in pro-coagulant as well as in the anti-coagulant pathway. Indeed, almost all the elements of clotting system are affected by liver disease, with an unstable haemostatic balance as a result. Platelet count is often reduced, as a consequence of hypersplenism, less frequently for defective production of thrombopoietin and growth factors, or the presence of anti-platelet antibodies, or folic acid deficiency, or drug toxicity^9^,^10^. Moreover, in the cirrhotic patients the platelets seem to suffer a kind of dysfunction, probably for the influence of endothelial inhibitory products (i.e. nitroxyd and prostacyclin), increased levels of von Willebrand factor, alterations of GpIb protein, and tromboxane A2 deficiency^11^. Thus, in cirrhotics the platelet dysfunction seems to be mostly restricted to the adhesion to sub-endothelium, but further studies are needed to confirm these data^11^–^13^.

Therefore, primary haemostasis is affected by altered platelet dysfunction, due to reduced platelet adhesion and to endothelial dysfunction; nevertheless, the ratio between low and high molecular weight subunits of von Willebrand factor is reduced. This is due to the presence of higher levels of high molecular weight subunits, with a capacity of adhesion counteracting the platelet dysfunction^11^.

As regards secondary haemostasis, the process of γ-carboxylation of factor II, VII, IX and X production is impaired in the patients with chronic liver disease. Indeed, liver storage of vitamin K is poor and, in case of cholestatic disease, its absorption is reduced. In contrast, factor VIII/von Willebrand factor ratio is always increased^15^. Moreover, fibrinogen levels appear quite stable in patients with stable liver disease, but they are sensibly lower in advanced stages or in acute liver failure. Dysfibrinogemia and decreased levels of factor V and XIII are common findings too.

**Figure 1.**
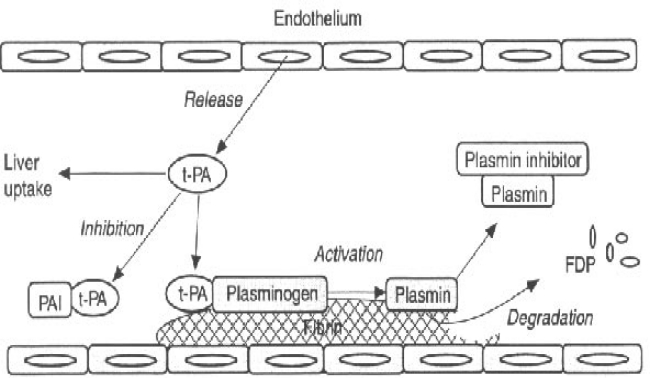
Schematic overview of the fibrinolytic system

As previously discussed, the alteration of pro-thrombotic mechanism coexists with decreased levels of natural anticoagulant factors, such as protein C and S, antithrombin and heparin cofactor II, all synthesized by liver^16^,^17^. At the same time, fibrinolysis may be altered (Figure 1). Plasminogen, α2-antiplasmin, α2-macroglobulin, and thrombin activatable fibrinolysis inhibitor (TAFI) levels may be reduced during acute or chronic liver disease, while tissue plasminogen activator (t-PA) levels are usually increased. Moreover, urokinase plasminogen activator (u-PA) and its receptor are increased but, during the progression of liver disease, plasminogen activator inhibitor 1 (PAI-1) production becomes prominent, even if at a lower rate in respect to those of t-PA. Thus, except in acute liver failure, when the increased PAI-1 may compromise fibrinolytic activity, the whole of these modifications predispose to hyperfibrinolysis.

Frequently, laboratory investigations may suggest a disseminated intravascular coagulation (DIC), but a relatively stable platelet count, high levels of factor VIII and a weak tendency to thrombin generation make the haemostatic state of cirrhotics different from DIC. The relative integrity of other organs confirms this opinion, even if signs of microthrombosis can be detected. Thus, this condition has been defined as “accelerated intravascular coagulation and fibrinolysis” (AICF), because of the high tendency to fibrinolysis and the resemblance with DIC^18^. The main feature of AICF is formation of a fibrin clot more susceptible to degradation by plasmin, due to high levels of t-PA, to inadequate release of PAI-1, and to reduced synthesis of α2-antiplasmin. Using specific laboratory assays, AICF can be detected in about 30% of cirrhotics, more frequently in patients with advanced liver failure^18^–^22^.

This complex system may also be perturbed, i.e. by renal insufficiency or bacterial infections, providing an increased predisposition to bleeding^22^–^24^.

Liver is also able to synthesize some coagulation inhibitors and to clear inactivated clotting factors and fibrinolytic enzymes. A study showed in rabbits that I^125^ marked thrombin aggregated to antithrombin was quickly removed from circulation by liver^14^, and a similar mechanism has been recognized for the complex heparin cofactor II – thrombin^26^–^28^. Apparently, liver has only one mechanism of uptake, which is based on a unique receptor, the low density lipoprotein receptor related protein (LRP); probably, vitronectin takes part to the process^29^. It is also notable that liver uptake works optimally only in presence of the enzyme aggregated to its target, but not in presence of the native or clivated enzyme. This mechanism may play a pivotal role in maintaining a normal haemostasis, by removing from circulation activated proteins. In addition, because t-PA degradation takes place into liver too, cirrhotic patients have increased t-PA levels, with some effects on fibrogenesis. Indeed, the system t-PA-plasmin is involved in activation of transforming growth factor-β (TGFβ), a profibrogenic cytokine, which is essential in the pathogenesis of liver cirrhosis. This complex interaction may link coagulation impairment of chronic liver disease and fibrosis progression, until cirrhosis development. Wanless et al.^30^,^31^ demonstrated that microthrombi are frequently present within hepatic vessels of patients with liver cirrhosis and other studies showed a correlation between inherited thrombophilic conditions and severity and progression of liver disease^32^–^35^. Moreover, HCV infected haemophilic patients seem to have a slower disease progression, supporting the hypothesis that a hypercoagulant state may heavily worsen the natural history of liver disease^36^.

Mechanisms underlying this strict interaction are still unknown, but two different theories seem to be plausible: parenchymal extinction and activation of stellate cells, which are pericytes found in the perisinusoidal space. According to parenchymal extinction hypotesis, vascular microthrombosis, triggered by necroinflammation, may cause liver ischemia and infarction, with loss of hepatic tissue replaced by fibrotic tissue; cirrhosis will result from the confluence of these microfibrotic areas. Alternatively, thrombin may induce fibrogenesis both directly, activating specific receptors on stellate cells, or indirectly, stimulating release of platelet-derived growth factors (PDGF) by platelets and promoting post-translational activation of TGFβ, an important mediator of fibrogenesis^37^–^40^. Thus, an excessive thrombin production my lead to hepatic fibrogenesis and cirrhosis; it is notable that stellate cells activation is, probably, not distincted by parenchymal extinction, confirming the role of coagulation in liver fibrosis progression^41^.

Finally, fibrinolysis is also affected by liver disease, since all proteins involved in fibrinolysis, except for tissue plasminogen activator (tPA) and plasminogen activator inhibitor 1 (PAI-1), are synthesized in the liver^41^.

In conclusion, cirrhotic patients seems to have a tendency to hyperfibrinolysis^43^, due to reduced levels of α2-antiplasmin, histidine-rich glycoprotein and factor XIII^44^–^49^; in contrast, t-PA levels are increased^15^,^51^–^56^ and those of PAI-1 are variable in each patient^16^,^17^,^52^. Recently, a great importance has been given to TAFI, an enzyme that inhibits fibrinolysis, postulating that its deficiency in cirrhotic patients may favor fibrinolytic pathway in cirrhotic patients^57^,^58^. However, this theory has not been fully confirmed^59^ Probably, this wide variability depends by a difference in laboratory assays. However, it must be remembered that infections and physiologic stress increase fibrinolytic activity through the release of tPA; also, ascitic fluid has a fibrinolytic activity, mostly related to its reabsorption in systemic circulation^60^,^61^. Thus, there are still concerns regarding the real fibrinolytic capacity of cirrhotic patients; laboratory investigations capable to evaluate global pro and anti fibrinolytic activity, rather than any single component, are missing. In the future, further studies should look after the validation of more reliable tests, to be employed in clinical practice.

## Coagulation Tests in Liver Cirrhosis:

Abnormal haemostatic tests and bleeding episodes are strictly associated in patients with liver cirrhosis, and it is a widely accepted opinion that the former may be the cause of the latter. Therefore, clinicians use to routinely monitor coagulation parameters and to correct any diagnosed alteration, to avoid the occurrence of complications after procedure at risk of bleeding (i.e. hepatic biopsy). As previously discussed, liver plays a primary role in the homeostasis of coagulative and fibrinolytic system; patients affected by chronic liver disease may benefit of this strict follow up to recognize early a possible and dangerous dysregulation. However, this tendency has been called into question, reassessing the value of tests used in clinical practice in predicting the real risk of bleeding and the suitability of possible therapeutic strategies^56^. It is well known that cirrhotic patients impairment in primary haemostasis is imputable to platelet quantitative and qualitative defects. They could be recognized by specific tests, like platelet count, aggregometry and cytofluorimetry, or by measurement of cutaneous bleeding time, prolonged in about 40% of cirrhotics^62^. However, whether this alteration has or not a clinical relevance is still questionable. Some authors demonstrated that a prolonged bleeding time (more than 12 minutes) is associated to an increased risk of hemoglobin reduction, after liver biopsy^63^. Regarding Von Willebrand factor dysfunction present in chronic liver disease, some experimental tests have recently been performed in condition mimicking *in vivo* blood flow circulation; it has been demonstrated that higher von Willebrand factor levels may compensate functional defect. These data confirm that since a prolonged bleeding time is not due to a mild thrombocytopenia or to a thrombocytopathy, it may be of scarce utility in predicting risk of bleeding in cirrhotic patients.

Liver cirrhosis is also characterized by reduced synthesis of the most part of clotting factors, recognizable by measuring blood concentration of each of them or, indirectly, by testing prothrombin time (PT) and activated partial thromboplastin time (aPTT). However, commonly PT and aPTT are weakly related to bleeding after risky procedures in cirrhotic patients. International normalized ratio (INR), derived from PT standardization, is one of the parameters evaluated in MELD scoring system, and is used for prognostic assessment and thus in transplantation to ensure organ allocation. Interestingly, since INR is used to test coagulation in patients on oral anticoagulants, the international sensitivity index (ISI) values for thromboplastin used to convert PT into INR derives by testing plasmas from this kind of patients, a very different condition from liver cirrhosis. Therefore, some authors reassessed INR values creating a different ISI index, using plasma from cirrhotic patients (instead of patients on oral anticoagulants) against the WHO international standard to provide calibration^64^. As a result, the INR obtained with different thromboplastins were not significantly different in cirrhotic patients, in contrast to patients on oral anticoagulants. Since INR is an important parameter of MELD scoring system and may alter the correct evaluation of patients prognosis, especially those candidates for transplantation, it could be considered whether manufacturers of commercial thromboplastins and point-of care coagulation monitors should provide two ISI values, one of which valid for cirrhotic patients^64^.

Probably, PT and aPTT are inadequate to investigate coagulation as it takes place *in vivo*, especially in presence of complex alterations of the pro- and anticoagulant system, such as chronic liver disease. Moreover, natural anticoagulants have to be activated to gain its function. *In vivo,* the activation of protein C is mediated by thrombin and its endothelial receptor thrombomodulin. Antithrombin too has to be activated by glycosamminoglicanes, like heparansolfate, localized on endothelial surface. Therefore, in vitro samples of PT and aPTT evaluation, free of thrombomodulin and glycosamminoglicanes, can only partially evaluate the sole function of procoagulant factors, excluding thrombin inhibition by anticoagulant ones. In synthesis, these samples are inadequate to evaluate defects in anticoagulation or the global mechanism and the complex interactions between pro- and anticoagulants.

Recently, haemostatic blood capacity of cirrhotic patients has been compared to that of healthy subjects, sampling the endogenous thrombin potential (ETP)^65^,^66^. It has been realized using the so called “thrombin generation test”, which is performed adding to plasma poor of platelet a small quantity of recombinant tissue factor and exogenous phospholipids, as platelet substitutes, to trigger coagulation; thrombomodulin may or not be added to activate protein C. The amount of generated thrombin is monitored using a fluorogenic substrate. Results are expressed by a curve, relating thrombin concentration to time; it is characterized by the lag phase, the peak of thrombin, the time to peak, and the area under the curve, which is called the “endogenous thrombin potential” (Figure 2). Tripodi et al.^21^,^68^ showed that in cirrhotic patients thrombin generated without addition of thrombomodulin was reduced, because of the reduced hepatic prothrombin synthesis. However, in the presence of thrombomodulin, their thrombin generation test was similar to healthy subjects, confirming the existence of such a balance between reduction of procoagulant (prothrombin) and anticoagulant (protein C) factors. The principal limit of this study was the use of plasma poor in platelet, differently from what happens *in vivo*. Thus, the same authors performed the thrombin generation test using platelet-rich plasma^13^; in the absence of thrombomodulin and with a standardized platelet count, cirrhotic patients generated a lower amount of thrombin in respect to controls. After the addition of thrombomodulin, this difference became inconsistent when platelet count was standardized, while, if the real patient platelet count was considered, thrombin generation by cirrhotics was decreased. In particular, the amount of thrombin was the more scarce the lower was platelet count. According to these results, thrombin generation test seems, currently, the most accurate in sampling the whole activity of pro- and anticoagulant factors, reproducing a system very close to *in vivo* conditions. However, even if this test may be useful in the future to predict the risk of bleeding in cirrhotic patients, it cannot consider other factors, such as infections and renal failure, which may influence *in vivo* haemostatic balance^67^,^68^.

## Predisposition to Bleeding or to Thrombosis?

Haemostasis in cirrhotic patients is the result of impairment in both pro and anticoagulant system, as previously discussed. This balance is hard to be maintained, sometimes producing bleeding, sometimes thrombosis and hypercoagulability. This condition has been compared to a “buffered” system, in which pro-coagulant tendency is counterbalanced by coagulation inhibitors and negative feedbacks^69^. In healthy subjects, plasma concentration of clotting factors is widely above the “security level”, necessary to ensure a correct coagulation; for this reason, physiologic, pathologic and stressful events have limited effects on haemostatic system, rarely determining bleeding. Similarly, patients affected by chronic liver disease may, in stable clinical conditions, maintain a normal haemostatic capacity, since the synthesis of only that part of clotting factors which exceeds “standard” level is reduced^21^,^70^. By the way, when this equilibrium is perturbed by extrinsic factors, such as infections, frequently the result is occurrence of bleeding or development of thrombosis^25^.

## Hypercoagulability and Liver Cirrhosis:

### Risk of Thrombosis:

Generally, a hypercoagulable state may manifest in both macrovascular or microvascular system. Microvascular thrombotic disease is often difficult to recognize and, for this reason, underdiagnosed. For cirrhotic patients, the principal trigger may be the presence of either a transient or chronic disequilibrium in haemostatic system, i.e. caused by infections, which leads to a loss of endothelial barrier function and activation of coagulation cascade with consequent thrombin generation. Moreover, vasoconstrictive and proliferative signals may favor the development of a plexogenic arteriopathy^71^. We already discussed how this mechanism may promote hepatic fibrogenesis and fibrosis progression to cirrhosis, influencing the natural time course of liver disease, but this microvascular thrombotic lesions are also involved in the pathogenesis of liver disease complications. Portopulmonary hypertension, alteration of vascular permeability in spontaneous bacterial peritonitis and renal dysfunction in type 2 hepatorenal syndrome probably recognize the microvascular disease as a common etiology^72^,^74^. Macrovascular thrombotic disease, instead, is more easily recognizable in cirrhotic patients, expressing with deep non-splanchnic venous thrombosis, pulmonary embolism and, frequently, portal vein thrombosis (PVT)^75^.

PVT has often a multifactorial etiology, in general population. A practical distinction between local and systemic conditions may be useful to recognize patients at high risk of PVT development (Table 1 and 2)^76^. Liver cirrhosis holds a central position among all other causes of PVT development, together with local inflammation or tumors^77^,^78^. PVT prevalence in cirrhotic patients is reported to be 0.6–16%, the highest among candidates for liver transplantation and of about 6.5% in patients with hepatocellular carcinoma at the moment of diagnosis^76^. It is notable how, in patients affected by chronic liver disease, inflammatory conditions or hepatocellular carcinoma may often coexist, contributing to PVT development. Furthermore, in presence of hepatic tumor, PVT incidence rises to 10–40% in cirrhotic patients^79^. Porto-systemic shunting, liver transplantation, ablative therapy for HCC or intra abdominal fine needle aspiration may represent other triggers for PVT development^80^. Apart from local risk factors, systemic disorders, such has, myeloproliferative neoplasms (MPN) and prothrombotic conditions, were reported to be responsible of about 40–60% of PVT in general population^77^,^81^. Factor V Leiden and protein C deficiency seems to be the most frequently involved; however, the role of protein C and protein S deficiency is difficult to be evaluated during anticoagulant therapy and is still to be confirmed^82^. Increased levels of factor VIII seems to be related to an increased risk of PVT development too^83^–^85^; in particular, cirrhotic patients with plasmatic levels of factor VIII beyond the 66^th^ percentile (129 IU/dL) are reported to have a 6-fold greater risk of PVT development^86^. However, the presence of a congenital prothrombotic disorder has always to be taken in account, even in cirrhotic patients. According to literature, prothrombin gene mutation seems to be the most frequent thrombophilic disorder among cirrhotics with PVT^87^–^91^.

Amitrano et al. also showed that prothrombin G20210A mutation is strongly related to PVT development in cirrhotic patients, even in absence of hepatocellular carcinoma^92^. In contrast, the question of anticardiolipin antibodies positivity is still discussed, since, although it is a frequent finding of laboratory investigations, it is influenced by infections and often considered as an indirect manifestation of liver damage^92^–^96^. This and other doubts are supported by the low estimated prevalence of genetic prothrombotic conditions among cirrhotic patients. Moreover, it is always hard to distinguish between hereditary or acquired deficiency of coagulation proteins, because of their plasmatic concentration is influenced by liver function impairment^97^. For all these reasons, to discover a potential genetic alteration in these subjects is often difficult and may lead to diagnostic mistakes^98^. Several studies tried to provide an alternative method, cheaper and easier to be performed than genetic screening. Some authors proposed the ratio of PS or PC or AT to [(factor II + factor X)/2] could furnish a sufficiently reliable overview of patient haemostatic alterations. A genetic thrombophilic condition is highly probable in case of a result lower than 70%^80^. Finally, familial screening is the more reliable tool to confirm the inborn nature of deficiency; indeed, such defects are inherited in a dominant manner, so their presence is proven by detecting the natural anticoagulant deficiency in at least one first-degree sibling. On the hand, the finding of normal levels of natural anticoagulants in both parents renders diagnosis unlikely.

MPN represent another common cause of PVT development in the general population^87^,^99^,^100^. In the Western Countries, 58% of idiopathic PVTs are associated to a latent MPN^101^,^102^. A simple method to investigate an underlying predisposition to MPN in PVT patients is screening the V617F point mutation in the gene encoding tyrosine-protein kinase JAK2^103^–^107^. Recently, it has been reported the presence of the JAK2 V617F gene mutation in about 17–35% of PVT patients without a known underlying disease^108^–^110^; however the prevalence among cirrhotic PVT patients is actually unknown.

In a recent study, Zocco et al.^111^ showed that portal flow velocity is strongly predictive of PVT development. In particular, 47.8% of patients with a portal flow velocity lower than 15 cm/sec at Doppler Ultrasound developed PVT, in contrast to only 2% of those with a measured velocity above 15 cm/sec. Probably, the reduced removal of thrombin due to blood stagnation, together with reduced levels of circulating anticoagulants, may produce the local conditions predisposing to PVT. This study opens new interesting perspectives, considering also that evaluation of portal flow velocity needs only a Doppler Ultrasound measurement, in clinical practice. However, further studies are necessary to confirm these data and to identify other factors helpful to recognize patients at high risk of PVT development^112^.

## Risk of Bleding and Liver Cirrhosis:

Bleeding manifestations are usually common during the natural course of chronic liver disease^42^, most of them not life threatening (i.e. bruising, petechiae, purpura, nose bleeds, and bleeding after dental extractions). The contribution of a defective haemostasis has been already evaluated in this review^113^,^114^; however, it is certain that the increasing in portal venous pressure is the most important trigger to bleeding. This hemodynamic alteration is not only confined to splanchnic vascular bed but, rather, it is a complex clinical syndrome affecting several systems and organs^115^. Hypervolemia, increased cardiac index, hypotension, decreased systemic vascular resistance and renal vasoconstriction configure the so called “effective hypovolemia”, which is part of the hyperkinetic syndrome often associated to portal hypertension; it is on the basis of ascites, hepatorenal syndrome, cirrhotic cardiomyopathy, hepatopulmonary syndrome and portopulmonary hypertension development and also responsible for hypersplenism-related pancytopenia and skin alterations (warm skin, palmar erythema, spider angiomas)^116^–^121^. However, bleeding from gastrointestinal tract is the most frightening manifestation of portal hypertension. Splanchnic vasodilation, decreased responsiveness to vasoconstrictors, neo-angiogenesis and formation of portal-systemic collaterals (gastroesophageal varices, portal hypertensive gastropathy and colopathy) are common findings in cirrhotic patients with portal venous pressure above 10 mmHg^129^–^131^. It is extimated that about 5–10% of cirrhotic patients per year develop new varices^125^–^129^, while 30–40% of them present varices at the time of diagnosis^123^,^128^,^129^. Variceal progression is associated to decompensation, high MELD or Child Pugh score, alcoholic etiology of cirrhosis and presence of red wale signs^130^–^131^. Bleeding incidence is reported about 4% per year, 15% in patients with medium-large varices; other risk factors are Child Pugh class, presence of red marks, hepatic venous pressure gradient (HVPG) above 12 mmHg^129^,^131^,^133^,^134^. As previously discussed, the role of cirrhotic coagulopathy in favoring variceal bleeding is questionable. However, the more severe is liver dysfunction the more difficult is to control bleeding, and often more prone to recur^142^,^143^. Alterations in primary and secondary haemostasis typical of advanced liver disease has been postulated as the principal explication; by the way, as we previously discussed in this article, the complex balance in cirrhotics haemostatic system does not necessarily predispose to bleeding. Otherwise, fybrinolitic activity has been recognized as a good predictor of gastrointestinal hemorrhage^137^,^138^. Probably, in the course of bleeding, a hyperfibrinolytic state compromises clot stabilization and favor its disruption. Moreover, coagulation may be delayed for the lower levels of clotting factors due to a continuous consumption and platelet function seems to be impaired too^141^–^142^. Some authors reported a correlation between fibrinogen degradation products or D-dimer levels, t-PA activity and the risk of bleeding, but pointing out a relativity low specificity; thus their use in clinical practice should be careful and evaluated on a case by case basis^143^,^144^.

## Conclusions:

Viral hepatitis is a major health problem worldwide, being the principal cause of chronic liver disease, cirrhosis and hepatocellular carcinoma. Once cirrhosis develops, patients are subjected to all complications derived from hepatic dysfunction and development of portal hypertension.

Among all these complications, haemostatic alterations have been considered for long time the principal cause of bleeding in cirrhotic patients. However, the scarce correlation between episodes of bleeding and alterations in coagulation test have changed this opinion. The balance between hypo and hypercoagulation, maintained by deficiency of both pro- and anticoagulant factors and fibrinolytic system, may explain why these patients are not protected by either the risk of bleeding or of thrombosis development.

## Figures and Tables

**Figure 268.**
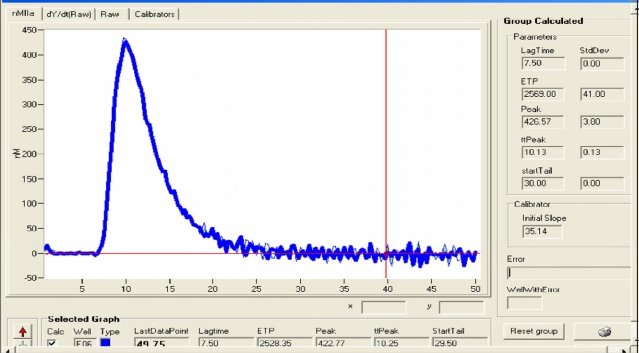
Thrombin generation curve (thrombogram). The endogenous thrombinic potential (ETP) corresponds to the area under the curve.

**Table 176.** Most frequent local risk factors for PVT

**LOCAL RISK FACTORS FOR PVT (70%)**
Cancer – Any abdominal organ Focal inflammatory lesions – Neonatal omphalitis, ombilical vein catheterization – Diverticulitis, Appendicitis – Pancreatitis – Duodenal ulcer – Cholecystitis – Tuberculous lymphadenitis – Crohn’s disease, Ulcerative colitis – Cytomegalovirus hepatitis Injury to the portal venous system – Splenectomy – Colectomy, Gastrectomy – Cholecystectomy – Liver transplantation – Abdominal trauma – Surgical portosystemic shunting, TIPS, – Iatrogenic (fine needle aspiration of abdominal masses etc.) Cirrhosis – Preserved liver function with precipitating factors (splenectomy, surgical portosystemic shunting, TIPS dysfunction, thrombophilia) – Advanced disease in the absence of obvious precipitating factors

**Table 276.** Frequent systemic risk factors for PVT

**SYSTEMIC RISK FACTORS FOR PVT (30%)**
Inherited: – Factor V Leiden mutation – Factor II (prothrombin) mutation – Protein C deficiency – Protein S deficiency – Antithrombin deficiency Acquired: – Myeloproliferative disorder – Antiphospholipid syndrome – Paroxysmal nocturnal hemoglobinuria – Oral contraceptives – Pregnancy or puerperium – Hyperhomocysteinemia – Malignancy
